# Supplementary material for: Scalable and sustainable synthesis of chiral amines by biocatalysis
Source: Commun Chem. 2025 Dec 12;8:403. doi: 10.1038/s42004-025-01783-w (PMC12717065; doi:10.1038/s42004-025-01783-w)
Supplement: Supplementary file 2 — Supplementary Information [file 42004_2025_1783_MOESM2_ESM.pdf]

**SUPPLEMENTARY INFORMATION**

# Scalable and Sustainable Synthesis of Chiral Amines by Biocatalysis

Matthew J. Takle,[a] David M. Maurer,[b] Philipp Staehle,[b] Joachim Dickhaut,[b] Christian Holtze,[b] Klaus Hellgardt,[c] King Kuok (Mimi) Hii[a,*]

[a] Department of Chemistry, Imperial College London, Molecular Sciences Research Hub, 82, Wood Lane, London W12 0BZ, U.K.

[b] BASF SE, Ludwigshafen 67056, Germany

[c] Department of Chemical Engineering, Imperial College London, Exhibition Road, South Kensington, London SW7 2AZ, U.K.

*mimi.hii@imperial.ac.uk

## 1. Materials and Characterisation

Unless otherwise stated, materials were purchased from commercial sources and used as received. All anhydrous solvents were dried over molecular sieves using solvent purification systems. 5 wt% Pd/γ-$Al_2O_3$ was purchased from Sigma and silicon carbide (SiC) was purchased from Fischer; both were sieved to afford samples with particle sizes of between 60–108 micron (Pd/γ-$Al_2O_3$) and 140-180 micron (SiC), before they were deployed in the packed beds. $^1$H and $^{13}$C NMR spectra were recorded on a 400 MHz Bruker AVANCE III HD spectrometer. Unless otherwise stated, $^1$H and $^{13}$C NMR chemical shifts are recorded of samples dissolved in $CDCl_3$. Chemical shift values were referenced to residual protic solvent peaks: $\delta_H$ = 7.26 ppm and $\delta_C$ = 77 ppm, respectively. Multiplicity is denoted as follows: s = singlet, b = broad, d = doublet, t = triplet, q = quartet, p = pentet, m = multiplet and coupling constants (*J*) are given in Hz. Infrared spectra (solid sample or thin film) were recorded on an Agilent Cary 630 FTIR fitted with a diamond ATR crystal. Chiral HPLC analysis was performed using an Agilent 1290 Infinity II instrument fitted with either an InfinityLab Poroshell 120 Chiral CF column (100 mm x 2.1 mm, 2.7 μm) using an isocratic solvent system of 80/20/0.3/0.2 MeCN/MeOH/acetic acid/triethylamine at 0.35 mL min$^{-1}$; or a Daicel Chiralcel OD (250 mm x 4.6 mm ID) column using an isocratic heptane/IPA (90/10) solvent system at 0.8 mL min$^{-1}$. Achiral HPLC analysis was performed using an InfinityLab Poroshell EC-C18 column (50 x 2.1 mm, 2.7 µm) using A (20 mM ammonium formate in $H_2O$) ramped against B (MeCN) (0.00 –2.00 min: 5% B, 2.00 – 6.00 min: 5–95 % B, 6.00–6.05 min: 95% B) at 0.5 mL min$^{-1}$. Chromatograms were recorded by a diode array UV-Vis detector, using 254 nm

absorbances for quantification. Achiral GC analysis was performed using a HP 6890 gas chromatogram equipped with a $H_2$ flame ionisation detector and an HP5 (5% phenyl methyl siloxane) Agilent column (30 m × 0.32 mm × 0.25 µm). The temperature ramp used was: 50 °C (hold 0.5 min), 50 – 150 °C (50 °C min-1 ), 150 - 300 °C (75 °C min-1 ), 300 °C (Hold 2 min). Specific terminologies, including conversion, selectivity, yield, are calculated below:

1. Conversion (%) refers to the amount of the amine precursor consumed during the (dynamic) kinetic resolution:

$$\text{Conversion (\%)} = \frac{[\text{Amine}]_{\text{Initial}} - [\text{Amine}]_{\text{Final}}}{[\text{Amine}]_{\text{Initial}}} \times 100$$ Supplementary Eqn. 1

2. Selectivity (%) refers to the amount of the desired product in the product mixture.

For the FTR:

$$\text{Selectivity (\%)} = \frac{[\text{Amine}]_{\text{Final}}}{[\text{Amine}]_{\text{Initial}}} \times 100$$ Supplementary Eqn. 2

For the DKR:

$$\text{Selectivity (\%)} = \frac{[\text{Amide}]_{\text{Final}}}{[\text{Amine}]_{initial} - [\text{Amine}]_{\text{final}}} \times 100$$ Supplementary Eqn. 3

3. Enantioselectivity (e.e.) refers to the optical purity of the chiral amine or chiral amide:

$$\text{e. e. (\%)} = \frac{[R]-[S]}{[R]+[S]} \times 100$$ Supplementary Eqn. 4

4. Space-Time-Yield (STY, mmol $L^{-1}$ $h^{-1}$) is calculated using the total amount of solvent, as shown in Eqn 1 in the manuscript. This can be converted to STY in mg $L^{-1}h^{-1}$ by multiplying by the molecular weight of the resolved amide.

**2. Screening of substrate scope, (Table 2)**

Using the reactor described in the previous report:[1] a PBR containing γ-$Pd/Al_2O_3$ (200 mg, sieved to 60–108 micron) was mixed with SiC (1 g, 180 micron), with a void volume of 1.05 mL. The reaction system was first primed with anhydrous toluene at 1 mL $min^{-1}$ for 10 min. The flow was then adjusted to 0.5 mL $min^{-1}$ while the reaction was heated. At 140 °C, the flow rate was set to 7 mL $min^{-1}$, until the in-line polarimeter showed a stable reading and set to zero. The inlet was then switched to the reagent line, containing a solution of the desired amine in toluene (82.5 mM). The reaction was monitored by the polarimeter, which was used to established when steady state had been achieved (indicated by a stable reading). The flow of the reactant was

maintained for a further 5 minutes at steady state, during which time 5 samples were collected (1 sample/min). The volume of sample collected will therefore depend on the flow rate i.e., 1/0.7/0.3 mL min-1 = 5 × 1/0.7/0.3 mL samples, respectively. The collected sample were diluted and subject to chiral HPLC and GC analysis to determine the e.e. and selectivity. If the e.e. and selectivity of a substrate remained high, the racemization was replicated at higher temperatures of 180 ºC, or 230 ºC, as necessary.

**3. Catalyst Screening**

HPLC vials pre-loaded with stir bars were charged with the desired catalysts (1 mol% metal loading with respect to the chiral amine), before they were placed into a 48-well Deck Screening Pressure Reaction (DSPR) plate, at randomly assigned positions, in duplicate. To each of the vial was dispensed 1 mL of an 82.5 mM stock solution of (*R*)-**1a** in anhydrous toluene, using a digital pipette. Two further HPLC vials were randomly distributed across the plate, one containing 1 mL of the stock solution and the other completely empty, to serve as controls. The vials were then covered by a Teflon sheet, followed by a silicone rubber vial cushion before a metal plate was gently screwed into position to secure the vials. The top plate of the DSPR was then fitted and screwed into place. The atmosphere in the DSPR was then exchanged by pressurising to 3.5 bar with $N_2$ before releasing the inert atmosphere. This was repeated three times, leaving the system under a $N_2$ atmosphere before the pressure was increased to 5 bar. The DSPR was heated on a heated deck on an Unchained Lab Junior reactor at 145 ºC, monitored by an internal thermocouple. Once the desired temperature had been reached, stirring at 200 RPM was initiated. After 1 hour the reactor was actively cooled to 15 ºC *via* the recirculatory chiller unit, before the pressure was released, and the top plate and vial covers removed. The DSPR was then transferred to a Pipetting Robot (Opentron OT-2), fitted with both 20 µL and a 1 mL pipette tips, for analytical sample preparation. A 20 µL aliquot from each reaction mixture was automatically extracted by pipette and dispensed into a new HPLC vial. This was followed by 1 mL of an anisole (internal standard) solution in MeOH (10 mM) stock solution, to achieve a 50-fold dilution. Each sample was analysed by (chiral) HPLC to determine e.e. and selectivity.

**Supplementary Table 1.** Catalyst loadings deployed for the catalyst screening campaign (Table S2).

| Entry | Group | Catalyst | Loading[a] (wt%) | Amount (mg) | Entry | Group | Catalyst | Loading[a] (wt%) | Amount (mg) |
|---|---|---|---|---|---|---|---|---|---|
| 1 | | Fe/$TiO_2$ | 1 | 4.61 | 10 | | Pd/$TiO_2$ | 1 | 8.78 |
| 2 | 8 | Ru/γ-$Al_2O_3$ | 4.84 | 1.72 | 11 | | Pd/C | 5 | 1.76 |
| 3 | | Ru/C | 4.82 | 1.73 | 12 | 10 | $Pd(OH)_2$/C | 5 | 1.76 |
| 4 | | Co/$TiO_2$ | 1 | 4.86 | 13 | | Pt/ γ -$Al_2O_3$ | 5.05 | 3.19 |
| 5 | 9 | Rh/ γ -$Al_2O_3$ | 5 | 1.70 | 14 | | Pt/$TiO_2$ | 1 | 16.1 |
| 6 | | Rh/$TiO_2$ | 1 | 8.49 | 15 | | Pt/C | 5 | 3.22 |
| 7 | | Ir/$TiO_2$ | 1 | 15.9 | 16 | | Cu/$TiO_2$ | 1 | 5.24 |
| 8 | 10 | Ni/$TiO_2$ | 1 | 4.84 | 17 | 11 | Au/$TiO_2$ | 1 | 16.3 |
| 9 | | Pd/ γ -$Al_2O_3$ | 5 | 1.76 | 18 | | Au/C | 1 | 16.3 |

[a]Metal content on the support. Not to be confused with catalyst loading (1 mol% with respect to chiral amine).

**Supplementary Table 2.** Results of screening group 8-10 metal catalysts in the racemization of (*R*)-**1a**.

| Entry | Group | Catalyst | e.e. (%)[a] | Selectivity (%)[b] | Entry | Group | Catalyst | e.e. (%)[a] | Selectivity (%)[b] |
|---|---|---|---|---|---|---|---|---|---|
| 1 | | Fe/$TiO_2$ | 100 | 100 | 10 | | Pd/$TiO_2$ | 7 | 15 |
| 2 | 8 | Ru/γ-$Al_2O_3$ | 100 | 95 | 11 | | Pd/C | 1 | 9 |
| 3 | | Ru/C | 100 | 98 | 12 | 10 | $Pd(OH)_2$/C | 31 | 38 |
| 4 | | Co/$TiO_2$ | 100 | 100 | 13 | | Pt/γ-$Al_2O_3$ | 28 | 68 |
| 5 | 9 | Rh/γ-$Al_2O_3$ | 99 | 99 | 14 | | Pt/TiO2 | 100 | 76 |
| 6 | | Rh/$TiO_2$ | 90 | 95 | 15 | | Pt/C | 14 | 30 |
| 7 | | Ir/$TiO_2$ | 100 | 92 | 16 | | Cu/$TiO_2$ | 100 | 100 |
| 8 | 10 | Ni/$TiO_2$ | 100 | 95 | 17 | 11 | Au/TiO2 | 100 | 94 |
| 9 | | Pd/γ-$Al_2O_3$ | 5 | 23 | 18 | | Au/C | 100 | 90 |

[a]Determined by chiral HPLC. [b]Determined by GC, see equation Supplementary Eq.2.

## 4. Catalyst Cartridge Preparation

### 4.1. Pd Packed Bed Reactor (FTR)

The end of a 1/4" OD SS column was sealed with a 1/4 -inch Swagelok fitting and SS frit, to which pre-sieved and pre-mixed 5 wt% Pd/γ-$Al_2O_3$ (200 mg, 60-108 micron) and SiC (450 mg, 180 micron) was added, before sealing in the same manner. Residence time was calculated from taking the difference between dry and wet weight of the column, to give a void volume of 0.33 mL (toluene ρ = 0.867 mg/mL) . This results in residence times of 20 seconds, 7 seconds and 4 seconds at 1 mL $min^{-1}$, 3 mL $min^{-1}$ and 5 mL $min^{-1}$ respectively.

## 4.2. Kinetic Resolution Packed Bed Reactor

A packed bed reactor was created by packing an SS column (1/2" OD. x 310 mm) with pre-mixed Novozym-435 (3 g) and SiC (20 g, 180 micron) sealed with 1/2" Swagelok fittings and SS frit. The column was mounted in a column heater (STH 585 Column Heater) and fitted in the Custom Flow Reactor where anhydrous toluene was passed through the packed bed at 1 mL $min^{-1}$. The packed bed column was then left for 0.5 h to allow the resin to swell. Residence time was calculated from taking the difference between dry and wet weight of the column, to give a void volume of 5 mL (toluene ρ = 0.867 mg/mL1 ). This corresponds to a residence time of 1 minute at a flow rate of 5 mL $min^{-1}$.

## 5. Racemisation/Kinetic Resolution Reactor Specifications

The HPLC pump (JASCO PU-980) used in the flow systems described below was fitted with a 10 mL pump head. The pump was maintained by regularly cleaning of the check values and pump components by sonication in methanol and checking flow rate of the reassembled system at different flow rates and temperatures. The detection range of the Jasco 2090 Flow Polarimeter was set to 5, Response set to Slow, and Gain set to x 1.

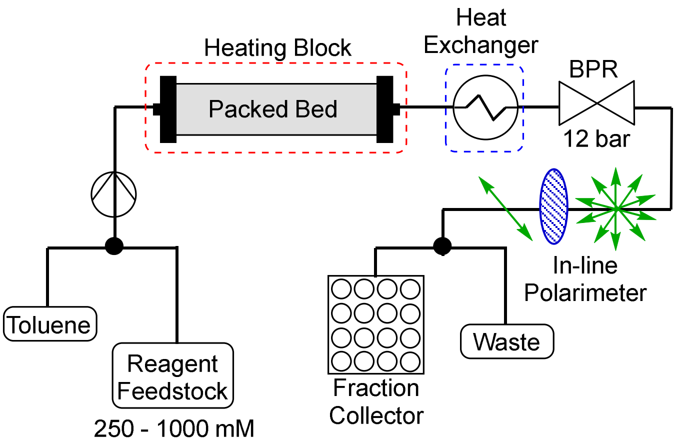


**Supplementary Fig. 1** Schematic of the FTR (packed bed = Pd catalyst) and KR (packed bed = Novozym-435) Flow System

A flow reactor system was constructed for the Flash Thermal Racemization (FTR) and Kinetic Resolution (KR) of chiral amines (Supplementary Fig. 1):

1) Solvents and amine solutions are delivered from RBF's containing inlet tubing and a nitrogen-filled balloon to keep the content under dry conditions (not shown). The inlet PFA tubing lines (1/16" OD, 1.59 mm ID, 50 cm) are connected to a three-way ball valve, which is connected to a JASCO UP-980 HPLC pump *via* (1/8" PFA tubing, 20 cm).

2) Using SS tubing (1/8” OD), the outlet of the pump is directly connected to a Swagelok T-piece, fitted with an in-line Omega digital pressure gauge. The pressure gauge was connected to the either the KR PBR housed within a column heater (STH 585 Column Heater), or FTR PBR *via* SS tubing (1/8” OD, 50 cm) which is housed inside a Thales Nano Phoenix Heating Block *via* SS tubing (1/8” OD, 50 cm).

3) The exit of the desired PBR was connected to SS tubing (1/8” OD, 50 cm) which passes through a custom-built cooling block milled to fit 1/8” OD SS tubing, powered by a 60 W Peltier thermo-electric cooling module and heatsink assembly (PiHut).

4) The exit of the cooling module is connected to a PFA tubing (1/8” OD, 20) to a back pressure regular (BPR), manually adjusted to 12 bar.

5) PFA tubing (1/16-inch, 80 cm) connects the BPR to a JASCO-2090 Polarimeter. Finally, PFA tubing (1/8” OD, 50 cm) connects the exit of the polarimeter to a two-way valve, which diverts the flowing solution either to a fraction collector (Spectrum Lab CD-2), or waste.

**6. Flash Thermal Racemization Chemoenzymatic Dynamic Kinetic Resolution Reactor Specifications**

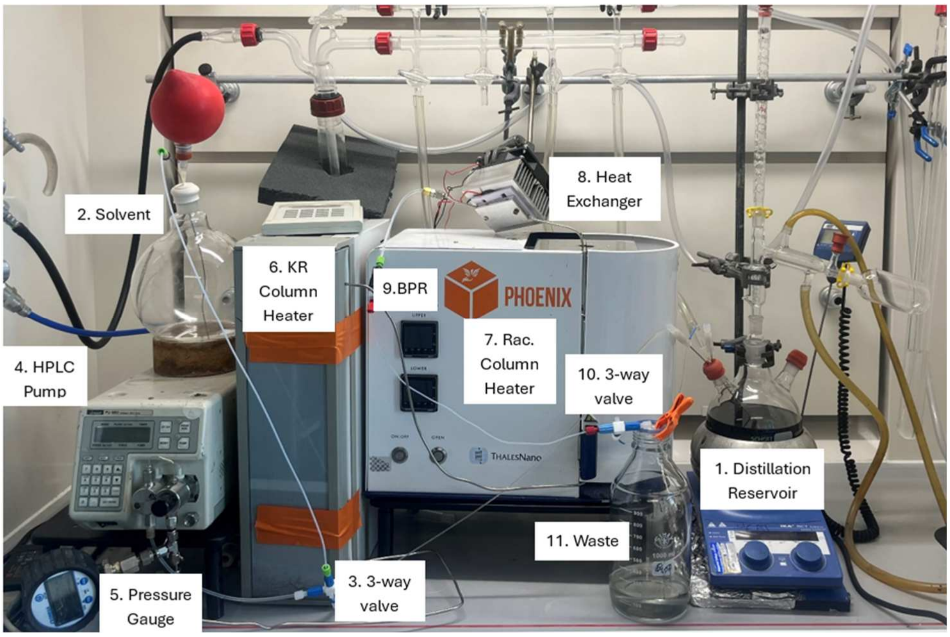


**Supplementary Fig. 2** FTR-CE-DKR Flow System

A flow reactor system was constructed for the Flash Thermal Racemization Chemoenzymatic Dynamic Kinetic Resolution (FTR-CE-DKR) of chiral amines (Supplementary Fig. 2):

1) Solvents and amine solutions are delivered from RBF's containing inlet tubing and a nitrogen-filled balloon to . The inlet PFA tubing lines (1/16" OD, 1.59 mm ID, 50 cm) are connected to a three-way ball valve, which is connected to a JASCO UP-980 HPLC pump *via* (1/8" PFA tubing, 20 cm).

2) Using SS tubing (1/8" OD), the outlet of the pump is directly connected to a Swagelok T-piece, fitted with an in-line Omega digital pressure gauge. The pressure gauge was connected to the Kinetic Resolution PBR *via* SS tubing (1/8" OD, 50 cm) housed within a column heater (STH 585 Column Heater). The exit of the KR PBR was connected to the Racemisation PBR *via* SS tubing (1/8" OD, 50 cm) which is housed inside a Thales Nano Phoenix Heating Block.

3) The exit of the Racemisation PBR was connected to SS tubing (1/8" OD, 50 cm) which passes through a custom-built cooling block milled to fit 1/8" OD SS tubing, powered by a 60 W Peltier thermo-electric cooling module and heatsink assembly (PiHut).

4) The exit of the cooling module is connected to a PFA tubing (1/8" OD, 20) to a back pressure regular (BPR), manually adjusted to 12 bar.

5) PFA tubing (1/8" OD, 50 cm) connects the BPR to a two-way valve, which diverts the flowing solution either to the reservoir which is heated to 70 °C to distil residual MeOH, or waste. The total reactor volume is approximately 10.52 mL, comprising of 5.19 mL tubing and 5.333 mL void volume in the PBR.

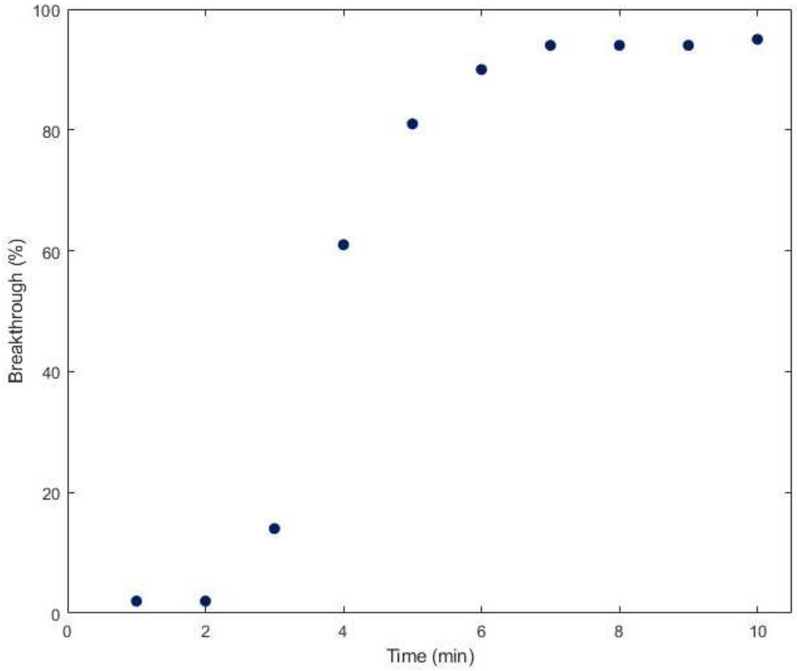


**Supplementary Fig. 3** Breakthrough Curve of **1c** (1M) through the CE-DKR flow system at 5 mL min$^{-1}$.

## 6. Procedure for the FTR (DoE)

The packed bed reactor was prepared as described in Section S3.1 and mounted in a Thales Nano Phoenix heating block. The flow reaction system primed by anhydrous toluene at 1 mL min$^{-1}$ for 10 minutes and maintained during heating. When the desired temperature was reached, the flow was then set to the desired rate (Supplementary Table 3), until the in-line polarimeter showed a stable reading and set to zero. The inlet was then switched to the reagent line, containing (*R*)–**1c**. The reaction was monitored by the polarimeter, which was used to established when steady state had been achieved (a stable reading). The flow of the reactant was maintained for a further 5 minutes at steady state, during which time 5 samples were collected (1 sample/min). The volume of sample collected will therefore depend on the flow rate i.e., 5/3/1 mL min$^{-1}$ = 5 x 5/3/1 mL samples, respectively. Analytical samples were prepared using a 50-fold dilution with a solution of anisole (10 mM, internal standard) in MeOH solution. The sample were subject to chiral and achiral HPLC analysis to determine e.e. and selectivity. 1-(3-methoxyphenyl)ethan-1-amine, **1c**: $^{1}$H NMR (400 MHz, $CDCl_3$) δ 7.26 (t, *J* = 7.7 Hz, 1H), 6.97 – 6.90 (m, 2H), 6.83 – 6.75 (m, 1H), 4.10 (q, *J* = 6.6 Hz, 1H), 3.83 (s, 3H), 1.54 (NH, s, 2H), 1.39 (d, *J* = 6.6 Hz, 3H). $^{13}$C NMR (101 MHz, $CDCl_3$) δ 159.86, 149.73, 129.58, 118.14, 112.14, 111.45, 55.29, 51.43, 25.74. IR: $\nu_{max}$/cm$^{-1}$ 3380, 2960, 1485, 1254, 1038. Chiral HPLC: InfinityLab Poroshell 120 Chiral CF: $t_R$ =2.12 min, $t_S$ = 2.34 min.

**Supplementary Table 3**. FTR DoE for the racemization of (*R*)-**1c**: 3 Factors and 2 responses.

| Entry | Whole Plots | Temp. (ºC) | Flow Rate (mL min$^{-1}$) | Residence Time (s) | Sol. Conc. (mM) | e.e. (%)[a] | Selectivity (%)[b] |
|---|---|---|---|---|---|---|---|
| 1 | 1 | 240 | 1 | 20 | 625 | 0 | 50 |
| 2 | 1 | 120 | 1 | 20 | 625 | 20 | 100 |
| 3 | 1 | 180 | 3 | 7 | 625 | 59 | 94 |
| 4 | 1 | 180 | 3 | 7 | 625 | 70 | 100 |
| 5 | 2 | 180 | 3 | 7 | 1000 | 36 | 93 |
| 6 | 2 | 120 | 1 | 20 | 1000 | 62 | 100 |
| 7 | 2 | 180 | 3 | 7 | 1000 | 42 | 100 |
| 8 | 2 | 240 | 5 | 4 | 1000 | 24 | 94 |
| 9 | 3 | 120 | 1 | 20 | 250 | 50 | 100 |
| 10 | 3 | 120 | 5 | 4 | 250 | 98 | 100 |
| 11 | 3 | 240 | 3 | 7 | 250 | 50 | 84 |
| 12 | 3 | 180 | 5 | 4 | 250 | 87 | 99 |
| 13 | 4 | 180 | 3 | 7 | 1000 | 60 | 96 |
| 14 | 4 | 240 | 5 | 4 | 1000 | 45 | 90 |
| 15 | 4 | 240 | 1 | 20 | 1000 | 2 | 16 |
| 16 | 4 | 120 | 5 | 4 | 1000 | 100 | 97 |
| 17 | 5 | 120 | 3 | 7 | 250 | 100 | 100 |
| 18 | 5 | 240 | 5 | 4 | 250 | 14 | 91 |
| 19 | 5 | 180 | 1 | 20 | 250 | 0 | 88 |
| 20 | 6 | 180 | 3 | 7 | 625 | 54 | 100 |
| 21 | 6 | 240 | 3 | 7 | 625 | 0 | 89 |
| 22 | 6 | 180 | 5 | 4 | 625 | 94 | 95 |
| 23 | 7 | 180 | 5 | 4 | 625 | 92 | 95 |
| 24 | 7 | 180 | 1 | 20 | 625 | 0 | 78 |
| 25 | 7 | 120 | 3 | 7 | 625 | 100 | 96 |

[a]Determined by chiral HPLC. [b]Determined by HPLC, see equation Supplementary Eq.2.

**Supplementary Table 4.** Repeated experiments (to check for catalyst deactivation).

| Entry | Temp. (°C) | Flow Rate (mL $min^{-1}$) | Residence Times (s) | Conc. (mM) | e.e. (%)[a] | Selectivity (%)[a] |
|---|---|---|---|---|---|---|
| 1 | 180 | 3 | 7 | 1000 | 36 (42) | 93 (100) |
| 2 | 180 | 5 | 4 | 250 | 86 (99) | 99 (99) |
| 3 | 240 | 5 | 4 | 1000 | 42 (48) | 90 (98) |
| 4 | 180 | 1 | 20 | 250 | 0 (0) | 90 (86) |
| 5 | 180 | 3 | 7 | 625 | 56 (52) | 100 (95) |

[a]Value in parenthesis corresponds to the result obtained by the repeat experiment.

## 7. Kinetic Resolution (DoE)

The flow reactor system was fitted with a packed bed of Novozym-435, prepared as described in Section S3.2. before it was primed by anhydrous toluene at 1 mL $min^{-1}$ for 10 minutes and maintained as heating commenced and the desired temperature was reached (Supplementary Table 5). The flow rate was then adjusted to the desired 5 mL $min^{-1}$ used in all experiments to align with the FTR. Once the in-line polarimeter showed a stable reading and set to zero, the inlet was switched to the reagent line containing a mixture of (*rac*)–**1c** and methyl 2-methoxyacetate (**3a**) at the desired concentrations and equivalents (Supplementary Table 5). When steady state had been achieved (indicated by a stable reading on the polarimeter), the reaction mixture was passed through the system for a further 5 minutes, during which time 5 reaction aliquots were collected every minute and analytical samples prepared as described above. 1-(3-methoxyphenyl)ethan-1-amine, **1c**: $^{1}$H NMR (400 MHz, $CDCl_3$) δ 7.26 (t, *J* = 7.7 Hz, 1H), 6.97 – 6.90 (m, 2H), 6.83 – 6.75 (m, 1H), 4.10 (q, *J* = 6.6 Hz, 1H), 3.83 (s, 3H), 1.54 (NH, s, 2H), 1.39 (d, *J* = 6.6 Hz, 3H). $^{13}$C NMR (101 MHz, $CDCl_3$) δ 159.86, 149.73, 129.58, 118.14, 112.14, 111.45, 55.29, 51.43, 25.74. IR: $\nu_{max}/cm^{-1}$ 3380 (1° N-H stretch), 2960 (Ar C-H), 1485 (C-H bend), (C-N stretch), 1254 (C-O stretch), 1038 (C-N, stretch). Chiral HPLC: InfinityLab Poroshell 120 Chiral CF: $t_R$ = 2.12 min, $t_S$ = 2.34 min. 2-methoxy-N-(1-(3-methoxyphenyl)ethyl)acetamide, **2c**: $^{1}$H NMR (400 MHz, $CDCl_3$) δ 7.26 (t, *J* = 7.9 Hz, 1H), 6.91 (d, 1H), 6.86 (s, 1H), 6.83 – 6.78 (m, 1H), 5.14 (dq, *J* = 14.9, 6.8 Hz, 1H), 3.92 (d, *J* = 15.3 Hz, 1H), 3.88 (d, *J* = 14.4 Hz, 1H), 3.80 (s, 3H), 3.40 (s, 3H), 1.50 (d, *J* = 6.9 Hz, 3H). $^{13}$C NMR (101 MHz, $CDCl_3$) δ 168.64, 159.92, 144.75, 129.85, 118.49, 112.55, 112.36, 72.06, 59.22, 55.34, 48.12, 22.01. IR: $\nu_{max}/cm^{-1}$ 3312, 2933, 1664, 1519. Chiral HPLC: OD-H Chiralcel: $t_R$ =9.27 min, $t_S$ = 14.3 min.

**Supplementary Table 5.** Kinetic Resolution DoE for the resolution of (*rac*)-**1c**: 3 factors and 2 responses

| Entry | Whole Plots | Temp. (ºC) | Acyl Donor (Equiv.) | Sol. Conc. (mM) | Amine e.e. (%)[a] | Amide e.e. (%)[a] | Conv. (%)[b] | E [c] |
|---|---|---|---|---|---|---|---|---|
| 1 | 1 | 20 | 2 | 250 | 54 | 99 | 35 | 200 |
| 2 | 1 | 70 | 2 | 250 | 82 | 99 | 45 | 200 |
| 3 | 2 | 70 | 2 | 1000 | 74 | 99 | 43 | 200 |
| 4 | 2 | 20 | 2 | 1000 | 34 | 99 | 26 | 200 |
| 5 | 3 | 20 | 0.5 | 250 | 24 | 99 | 20 | 200 |
| 6 | 3 | 70 | 0.5 | 250 | 44 | 99 | 31 | 200 |
| 7 | 4 | 70 | 0.5 | 1000 | 48 | 99 | 33 | 200 |
| 8 | 4 | 39 | 0.5 | 1000 | 36 | 99 | 27 | 200 |
| 9 | 5 | 45 | 1.25 | 625 | 50 | 99 | 34 | 200 |
| 10 | 5 | 70 | 1.25 | 625 | 58 | 99 | 37 | 200 |
| 11 | 6 | 20 | 1.25 | 625 | 34 | 99 | 26 | 200 |
| 12 | 6 | 45 | 1.25 | 625 | 46 | 99 | 32 | 200 |
| 13 | 7 | 45 | 2 | 625 | 58 | 99 | 37 | 200 |
| 14 | 7 | 45 | 2 | 625 | 60 | 99 | 38 | 200 |
| 15 | 8 | 45 | 1.25 | 250 | 62 | 99 | 39 | 200 |

[a]Determined by chiral HPLC. [b]conversion = $e.e._S/(e.e._S+e.e._P)$; $e.e._S$ = e.e. of recovered (*S*)-**1c** in the reaction mixture and $e.e._P$ = e.e. of recovered (*R*)-**2c** in the reaction mixture.[c]Selectivity factor for the kinetic resolution, $E = \ln[(1-c)(1-e.e._S)]/\ln[(1-c)(1+e.e._S)]$; where c = conversion.

## 8. Large scale FTR of *R* -1-(3-methoxyphenyl)ethan-1-amine, (*R*)-1c

The packed bed reactor was prepared as described in Section S3.1. and mounted in the ThalesNano Phoenix reactor. The flow reaction system was primed with anhydrous toluene at 1 mL min$^{-1}$ for 10 minutes while the PBR was heated to 230 ºC, with the outlet directed to waste. When the desired temperature was reached, the flow was increased to 5 mL min$^{-1}$, until the in-line polarimeter showed a stable reading and was then set to zero. The inlet was switched to the reagent line to deliver a 1 M solution of (*R*)–**1c**. Once the polarimeter reading confirmed breakthrough of the reagent solution, the outlet was switched to direct the product stream into the reservoir containing (*R*)–**1c**. Samples were taken from the reservoir every 10 minutes or 2 hours, for the 15 g or 150 g reactions respectively until full racemisation was achieved.

**Supplementary Table 6.** 15 g racemisation of *R* -1-(3-methoxyphenyl)ethan-1-amine, ***(R)*-1c** (Fig. 4).

| Time (min) | e.e. (%)[a] |
|---|---|
| 0 | 100 |
| 10 | 78 |
| 20 | 54 |
| 30 | 40 |
| 40 | 26 |
| 50 | 20 |
| 60 | 14 |
| 70 | 8 |
| 80 | 4 |

[a]Determined by chiral HPLC

**Supplementary Table 7.** 150 g racemisation of *R* -1-(3-methoxyphenyl)ethan-1-amine, ***(R)*-1c** (Fig 4).

| Time (h) | e.e. (%)[a] |
|---|---|
| 0 | 100 |
| 2 | 58 |
| 4 | 42 |
| 6 | 34 |
| 7 | 26 |
| 9 | 20 |
| 11 | 12 |
| 13 | 10 |
| 15 | 6 |

[a]Determined by chiral HPLC

**9. Large scale FTR-CE-DKR of *R*–2-methoxy-*N*-(1-(3-methoxyphenyl)ethyl)acetamide, (*R)*-2c**

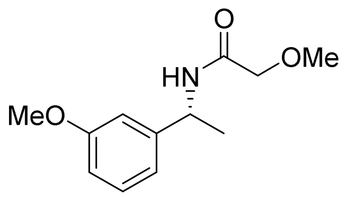


The flow reactor system (Supplementary Fig. 2) was fitted with the Novozym-435 (KR) and Pd/γ-$Al_2O_3$ (FTR) packed bed reactors, both prepared as described in Sections S3.1 and S3.2. The reactor was primed by anhydrous toluene at 1 mL $min^{-1}$ for 10 minutes, before the KR and FTR reactors were heated to 70 $^oC$ and 230 $^oC$, respectively. Once the desired temperatures were reached, the flow rate was then increased to 5 mL $min^{-1}$ for 5 minutes before the inlet was switched to the delivery a solution of (*rac*)–**1c** (100 g, 661 mmol) and methyl 2-methoxyaceate, **3a** (138 g, 2 equiv.) in toluene (661 mL, 1 M) from a 1L, 3-necked RB flask fitted with a distillation column. During the reaction, the RB flask was heated and stirred at 70 ºC to remove the MeOH by-product *via* azeotropic distillation. Aliquots of the reaction mixture (100 µL) were extracted from the reservoir every hour for HPLC analysis. After each extract, additional portions of toluene were added to the reservoir to compensate the loss of solvent, thus maintaining the total volume of the reaction mixture at 661 mL. After 14 h, the reaction reached 95% conversion and 90% selectivity (HPLC). The reaction mixture was cooled down, washed with 2 M HCl (3 x 50 mL), dried with $MgSO_4$, filtered and reduced in vacuo to yield (*R*)-**2c** as an orange oil (101 g, 68%, 99 % e.e.).

$^1$H NMR (400 MHz, $CDCl_3$) δ 7.26 (t, *J* = 7.9 Hz, 1H), 6.91 (d, 1H), 6.86 (s, 1H), 6.83 – 6.78 (m, 1H), 5.14 (dq, *J* = 14.9, 6.8 Hz, 1H), 3.92 (d, *J* = 15.3 Hz, 1H), 3.88 (d, *J* = 14.4 Hz, 1H), 3.80 (s, 3H), 3.40 (s, 3H), 1.50 (d, *J* = 6.9 Hz, 3H). $^{13}$C NMR (101 MHz, $CDCl_3$) δ 168.64, 159.92, 144.75, 129.85, 118.49, 112.55, 112.36, 72.06, 59.22, 55.34, 48.12, 22.01. IR: $\nu_{max}/cm^{-1}$ 3312, 2933, 1664, 1519. Chiral HPLC: OD-H Chiralcel: $t_R$ = 9.27 min, $t_S$ = 14.3 min.

**Supplementary Table 8.** Large scale FTR-CE-DKR of *R*–2-methoxy-*N*-(1-(3-methoxyphenyl)ethyl)acetamide, ***(R)*-2c** (Fig. 7).

| Time (h) | Conversion (%) | e.e. (%)[a] | Selectivity (%)[b] |
|---|---|---|---|
| 0 | 0 | 99 | 100 |
| 1 | 15 | 99 | 99 |
| 2 | 25 | 99 | 100 |
| 3 | 37 | 99 | 100 |
| 4 | 52 | 99 | 98 |
| 5 | 57 | 99 | 96 |
| 6 | 63 | 99 | 94 |
| 7 | 68 | 99 | 95 |
| 8 | 72 | 99 | 98 |
| 9 | 77 | 99 | 88 |
| 10 | 83 | 99 | 89 |
| 11 | 86 | 99 | 89 |
| 12 | 90 | 99 | 90 |
| 13 | 91 | 99 | 87 |
| 14 | 95 | 99 | 88 |

[a]Determined by chiral HPLC. [b]Determined by HPLC, see equation Supplementary Eq.3.

**10. Recovery of (*R*)-1-(3-methoxyphenyl)ethan-1-amine, (*R*)–1c, from (*R*)-2c.**

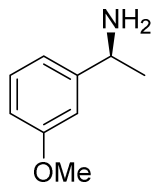


A solution of (*R*)-**2c** (10 g) in HCl:$H_2O$ (1:1 w/v, 90 mL) was refluxed with stirring for 16 h. The reaction mixture was allowed to cool to room temperature and basified with the addition of NaOH pellets. The neutralised amine was extracted from the aqueous solution with toluene (2 x 50 mL). The combined organic layer was reduced in vacuo, and the resulting crude material was distilled (140 ºC, 10 mbar) to afford *R*–**1c** as a colourless oil (5 g, 74 %, 99 % e.e.). $^1$H NMR (400 MHz, $CDCl_3$) δ 7.26 (t, *J* = 7.7 Hz, 1H), 6.97 – 6.90 (m, 2H), 6.83 – 6.75 (m, 1H), 4.10 (q, *J* = 6.6 Hz, 1H), 3.83 (s, 3H), 1.54 (NH, s, 2H), 1.39 (d, *J* = 6.6 Hz, 3H).

$^{13}C$ NMR (101 MHz, $CDCl_3$) δ 159.86, 149.73, 129.58, 118.14, 112.14, 111.45, 55.29, 51.43, 25.74. IR: $\nu_{max}/cm^{-1}$ 3380, 2960, 1485, 1254, 1038. Chiral HPLC: InfinityLab Poroshell 120 Chiral CF: $t_R$ = 2.12 min, $t_S$ = 2.34 min.

## 11. Analytical Data

### 11.1. Chiral HPLC

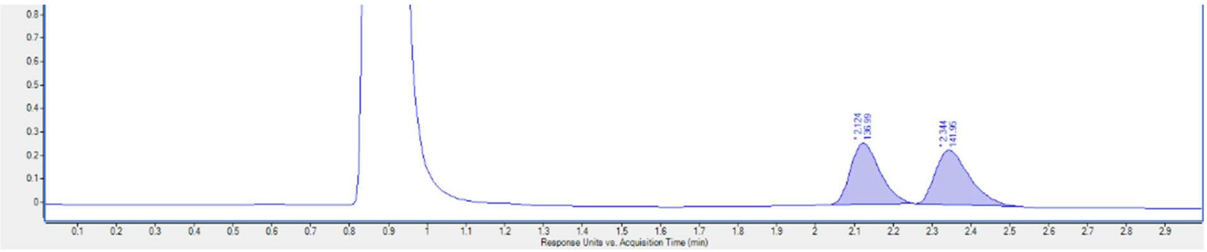


| Peak | RT | Area | Area Sum % | Height |
|---|---|---|---|---|
| 1 | 2.124 | 136.99 | 49.11 | 26.12 |
| 2 | 2.344 | 141.95 | 50.89 | 23.24 |

**Supplementary Fig. 4** Chiral HPLC chromatogram of 1-(3-methoxyphenyl)ethan-1-amine, (*rac*)–**1c**, (InfinityLab Poroshell 120 Chiral CF).

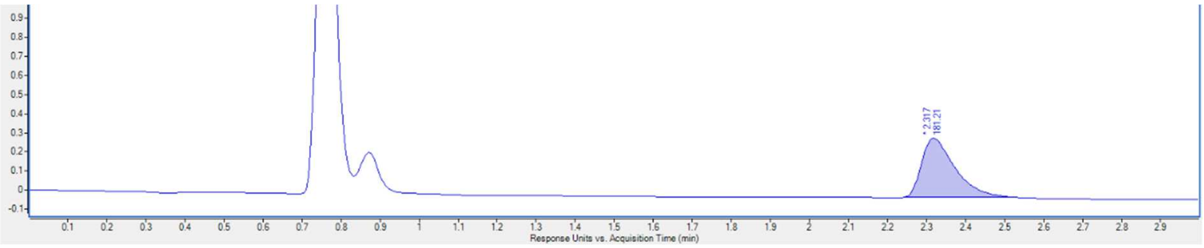


| Peak | RT | Area | Area Sum % | Height |
|---|---|---|---|---|
| 1 | 2.317 | 181.21 | 100 | 31.07 |

**Supplementary Fig. 5** Chiral HPLC chromatogram of (*R*)-1-(3-methoxyphenyl)ethan-1-amine, (*R*)–**1c**, (InfinityLab Poroshell 120 Chiral CF).

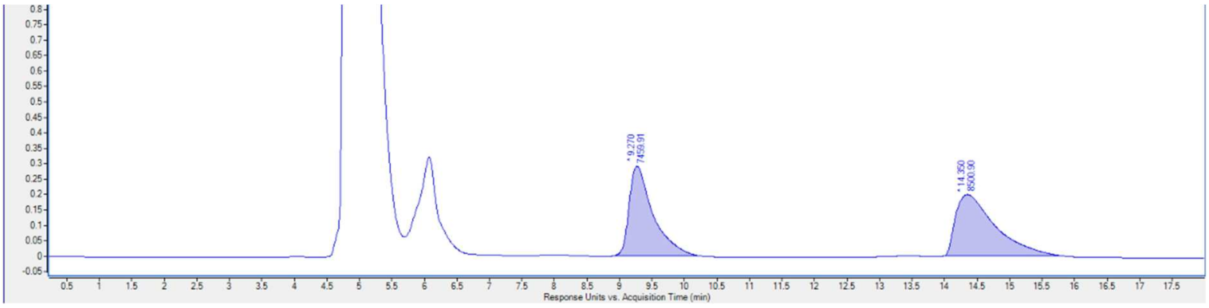

| Peak | RT | Area | Area Sum % | Height |
|---|---|---|---|---|
| 1 | 9.27 | 7459.91 | 46.74 | 290.24 |
| 2 | 14.3 | 8500.9 | 53.26 | 199.29 |

**Supplementary Fig. 6** Chiral HPLC chromatogram of 2-methoxy-N-(1-(3-methoxyphenyl)ethyl)acetamide, (*rac*)–**2c**, (Chiralcel OD).

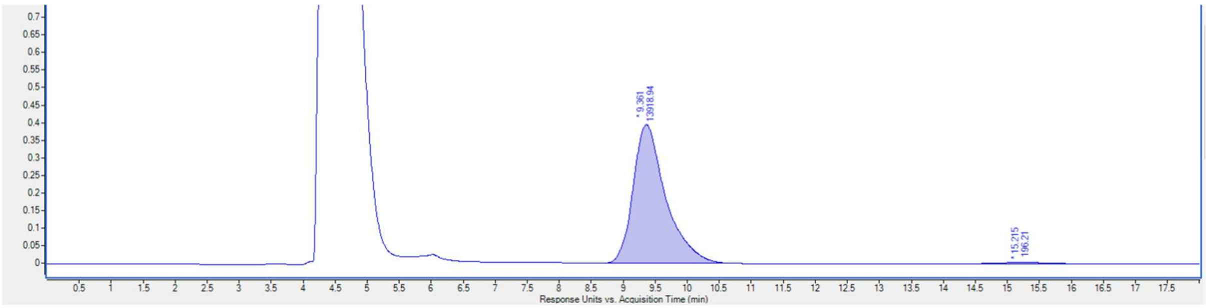

| Peak | RT | Area | Area Sum % | Height |
|---|---|---|---|---|
| 1 | 9.361 | 13918.94 | 98.61 | 393.62 |
| 2 | 15.215 | 196.21 | 1.39 | 4.15 |

**Supplementary Fig. 7** Chiral HPLC chromatogram of (*R*)-2-methoxy-N-(1-(3-methoxyphenyl)ethyl)acetamide, (*R*)–**2c**, (Chiralcel OD column)

## 11.2. NMR spectra

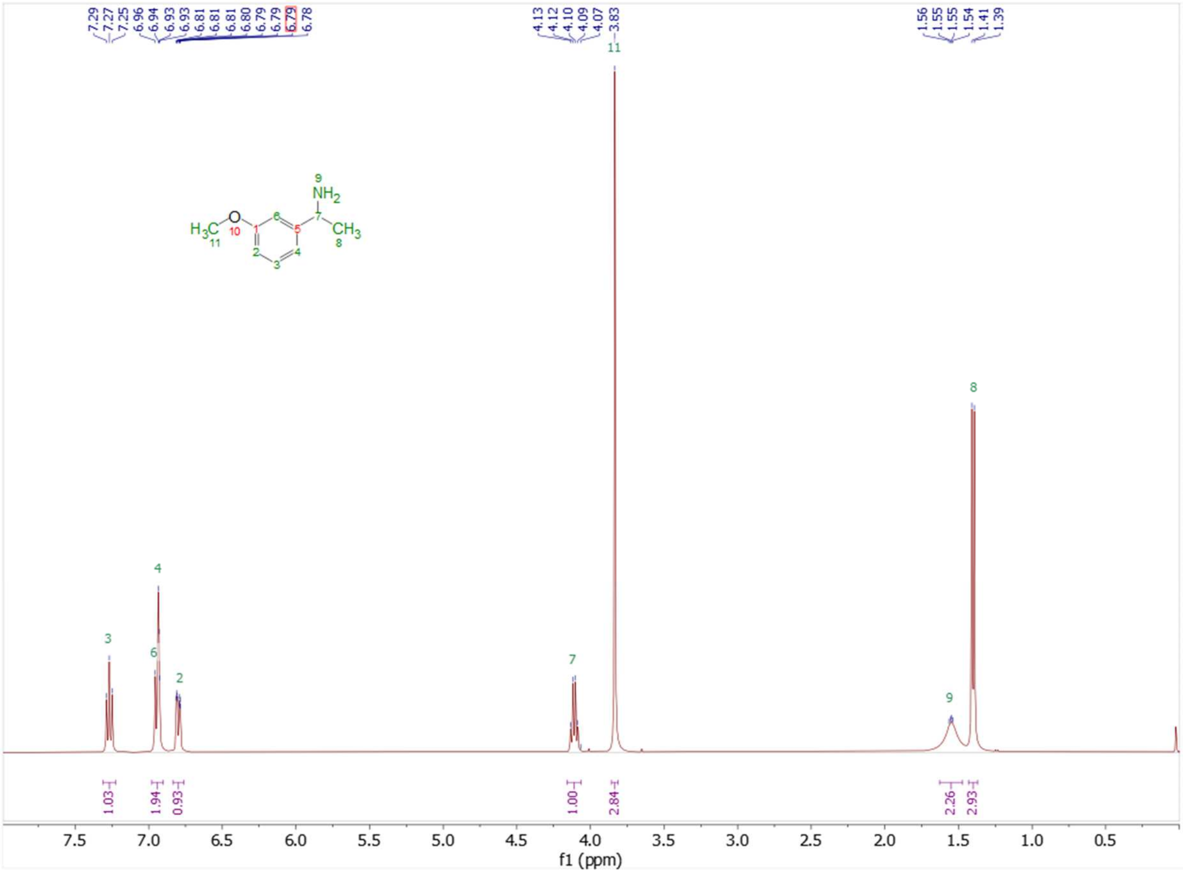


**Supplementary Fig. 8** $^{1}$H NMR, 1-(3-methoxyphenyl)ethan-1-amine, **1c**

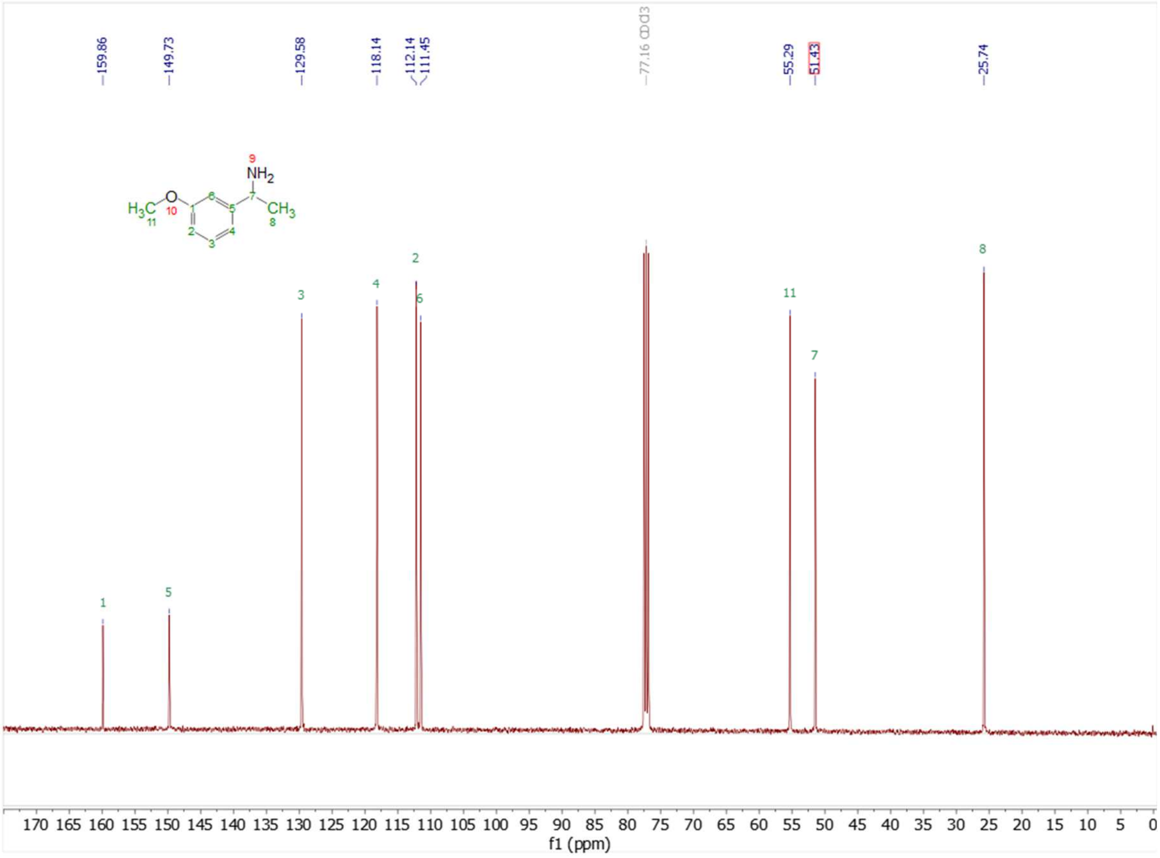


**Supplementary Fig. 9** $^{13}$C NMR, 1-(3-methoxyphenyl)ethan-1-amine, **1c**

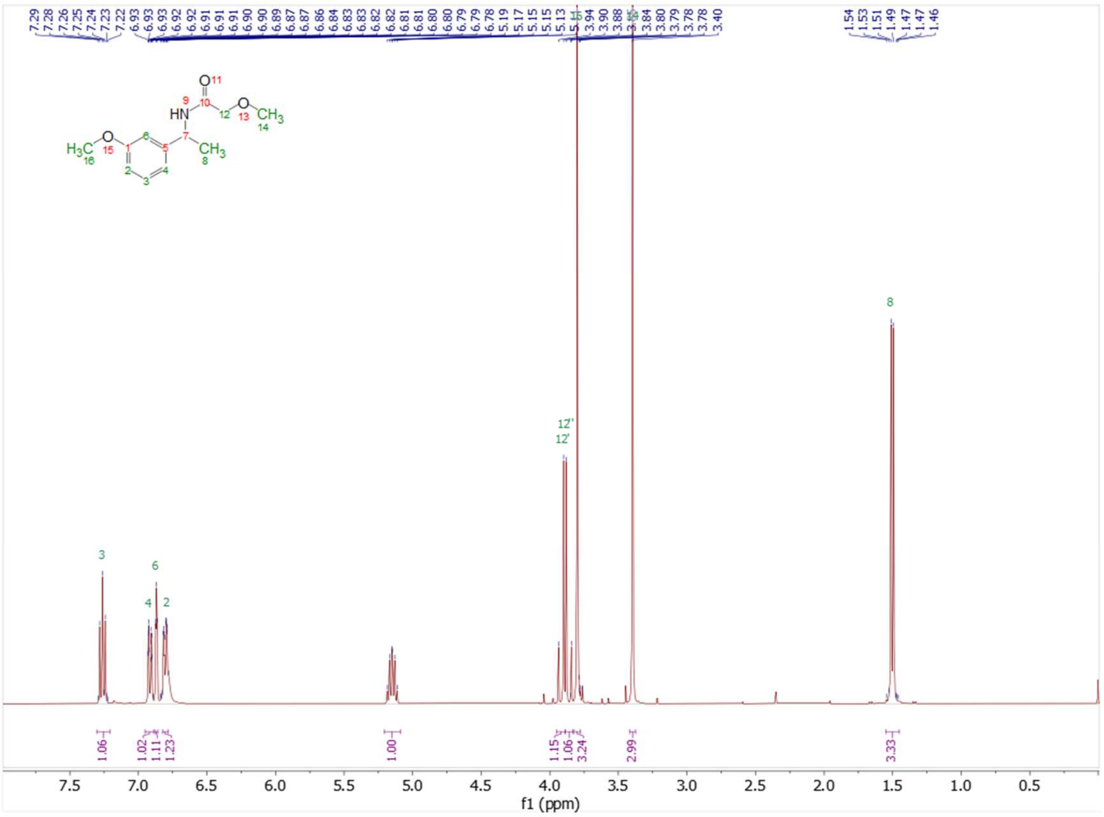

**Supplementary Fig. 10** $^{1}$H NMR, 2-methoxy-N-(1-(3-methoxyphenyl)ethyl)acetamide, **2c**

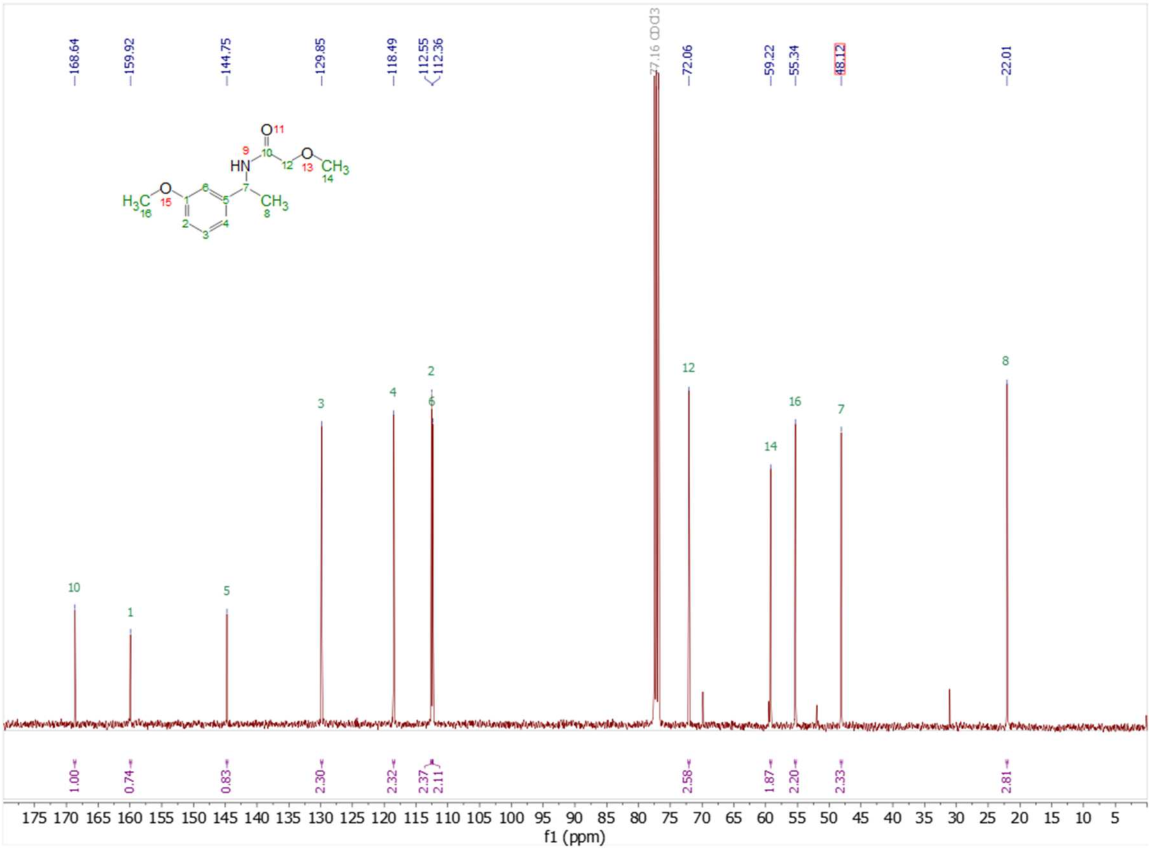

**Supplementary Fig. 11** $^{13}$C NMR, 2-methoxy-N-(1-(3-methoxyphenyl)ethyl)acetamide, **2c**

## 12. Supplementary Reference

1. Takle, M. J. *et al.* A Flash Thermal Racemization Protocol for the Chemoenzymatic Dynamic Kinetic Resolution and Stereoinversion of Chiral Amines. *ACS Catal*. **13**, 10541-10546 (2023).
